# Start-Up and Aeration Strategies for a Completely Autotrophic Nitrogen Removal Process in an SBR

**DOI:** 10.1155/2017/1089696

**Published:** 2017-12-13

**Authors:** Xiaoling Zhang, Fan Zhang, Yanhong Zhao, Zhengqun Li

**Affiliations:** ^1^School of Environmental Science and Engineering, Chang'an University, Xi'an 710064, China; ^2^Key Laboratory of Subsurface Hydrology and Ecological Effect in Arid Regions, Ministry of Education, Xi'an 710064, China

## Abstract

The start-up and performance of the completely autotrophic nitrogen removal via nitrite (CANON) process were examined in a sequencing batch reactor (SBR) with intermittent aeration. Initially, partial nitrification was established, and then the DO concentration was lowered further, surplus water in the SBR with high nitrite was replaced with tap water, and continuous aeration mode was turned into intermittent aeration mode, while the removal of total nitrogen was still weak. However, the total nitrogen (TN) removal efficiency and nitrogen removal loading reached 83.07% and 0.422 kgN/(m^3^·d), respectively, 14 days after inoculating 0.15 g of CANON biofilm biomass into the SBR. The aggregates formed in SBR were the mixture of activated sludge and granular sludge; the volume ratio of floc and granular sludge was 7 : 3. DNA analysis showed that Planctomycetes-like anammox bacteria and* Nitrosomonas*-like aerobic ammonium oxidization bacteria were dominant bacteria in the reactor. The influence of aeration strategies on CANON process was investigated using batch tests. The result showed that the strategy of alternating aeration (1 h) and nonaeration (1 h) was optimum, which can obtain almost the same TN removal efficiency as continuous aeration while reducing the energy consumption, inhibiting the activity of NOB, and enhancing the activity of AAOB.

## 1. Introduction

Biological nitrogen removal processes are generally used for the elimination of nitrogen from wastewater. However, wastewater with low carbon to nitrogen ratios (C/N), such as supernatants of anaerobic sludge digesters, landfill leachate, and special industrial wastewater, makes the conventional nitrification and heterotrophic denitrification processes more difficult [1, 2]. The discovery of the anaerobic ammonium oxidation (anammox) process has revolutionized the removal of nitrogen from wastewater that contains high amounts of nitrogen and has low C/N [3]. The anammox process involves the oxidation of ammonia into nitrogen gas, with nitrite serving as an electron acceptor in the absence of oxygen and organic carbon compounds [4]. Anammox is a promising process in which the costs related to aeration and external carbon addition are lowered by 60% and 100%, respectively, and which results in 90% less sludge compared to conventional nitrification-denitrification processes [5–7]. Several autotrophic nitrogen removal processes have been developed based on anammox, such as the two-stage SHARON-ANAMMOX process [8] and the one-stage CANON (completely autotrophic nitrogen removal via nitrite) [9, 10] and OLAND (oxygen-limited autotrophic nitrification-denitrification) [11] processes. One-stage processes combine two reactions that are catalyzed by two different microbial groups, aerobic ammonium oxidizing bacteria (AerAOB) and anaerobic ammonium oxidizing bacteria (AnAOB). Initially, AerAOB oxidizes NH_4_^+^ to NO_2_^−^ under aerobic conditions (i.e., partial nitrification), which is followed by AnAOB converting NH_4_^+^ and NO_2_^−^ into N_2_ [12]. CANON is one of the autotrophic nitrogen removal processes that maintain AerAOB and AnAOB in a single reactor by controlling low DO levels [13].

The long start-up period of the CANON process is the bottleneck for its application due to the very slow growth rate (doubling time is 11 d) and the low yield coefficient (0.13 g dry weight/g NH_4_-N oxidized) of AnAOB [4, 14, 15]. There are two strategies used to initiate the CANON process [16]. The first strategy is to inoculate an anammox reactor with nitrifying biomass and subsequently maintain the oxygen-limited conditions [13, 17]. The second is to operate a nitrifying reactor under oxygen-limited conditions in order to wash out NOB and later inoculate the AnAOB biomass [18, 19]. For the application of the CANON process, the strategy of initially inoculating universally nitrifying activated sludge, and then inoculating small amounts of AAOB in order to partially nitrify the reactor, was the most practical [20].

The CANON process can be developed in both biofilm and suspended systems. For suspended-growth systems, there are two challenges: biomass retention and the equilibration of different microbial group activities [21]. Granular sludge not only guarantees high biomass retention efficiency but also protects inner AAOB from dissolved oxygen [22]. Recent studies state that small and large aggregates play different functional roles in the nitrite-anammox process [21, 22]. It was reported that large type aggregates (>500 *μ*m) accounted for 68% of the AnAOB, whereas 65% of the nitrification potential was observed in the smaller aggregates (<500 *μ*m) in a granular reactor [21]. The aggregates formed in granular sludge reactors contain a mixture of flocs, small granules, and large granules [23–26]. Granular sludge can be defined as compact and dense aggregates with an approximately spherical external appearance that did not coagulate under decreased hydrodynamic shear conditions and that settle significantly faster than floc [27]. In comparison, floc is characterized by loose, permeable aggregates that are composed of microcolonies enmeshed in extracellular polymers [28]. In granular aggregates, AerAOB lies in the outer layer and AnAOB in the inner layer, while in floc, AerAOB were the dominant bacteria [22]. To achieve high nitrogen removal efficiency, a good balance of AerAOB and AnAOB activity is needed, which is related to the aggregate characteristics in the reactor.

In the CANON process, the continuous aeration mode was applied more often than the intermittent aeration mode. Lackner stated that continuous aeration is preferred under normal operating conditions, while intermittent aeration is primarily used during start-up or periods of low sludge activities [1]. It was also reported that the intermittent aeration strategy can obtain similar total nitrogen removal efficiency as the continuous aeration strategy, thereby saving energy consumption due to shorter aeration times [29], but the proper ratio of aeration to nonaeration time should be well considered.

In this study, partial nitrification was established in a sequencing batch reactor by carefully controlling the aeration, initially using activated sludge as seed sludge and then inoculating CANON biofilm in the SBR. This method achieved both partial nitrification and anammox in one single-stage reactor by reducing the aeration amount and altering the continuous and intermittent aeration amounts. This process was examined, the sludge characteristics were studied with SEM, and the polymerase chain reaction (PCR) technique was used to analyze the microbial community in the reactor. Additionally, the influence of aeration strategy on the CANON process was studied in batch tests.

## 2. Materials and Methods

### 2.1. Reactor Description

This study used a SBR with a working volume of 13 L. Dimensions of the SBR were the following: height of 545 mm, inner diameter of 174 mm, and height to diameter ratio of 3.13 : 1. Two or three cycles were performed each day during the whole experiment. One cycle consisted of a 20 min filling period, an aerating period (continuous or intermittent) of 11 h in 12 h cycle or 7 h in 8 h cycle, a 30 min settling period, a 5 min drawing period, and a 5 min idling period. The exchange volume was fixed at 50%, and a mass-flow controller was used to keep aeration constant. Two different aeration strategies were applied in this study, namely, continuous aeration and intermittent aeration, both characterized by the ratio (R) between the aerated and nonaerated duration times. The reactor was stirred at a rate of 700 rpm and maintained at a temperature of 30 ± 2°C.

### 2.2. Biomass and Synthetic Wastewater

In this study, the conventional nitrification sludge originated from the oxidation ditch of a municipal wastewater plant, was inoculated and developed to partial nitrification in the SBR, and was later inoculated with a small CANON biofilm biomass (0.15 g) to partial nitrification in the SBR.

The synthetic wastewater used in this study mainly contained NH_4_^+^ from NH_4_HCO_3_ (N source, C source, and buffer), NaHCO_3_ (supplementary C source and buffer), and phosphate from KH_2_PO_4_. The influent concentrations were sampled from the filing tank. The influent ammonium concentration was in the range of 100~300 mg/L and the pH was between 7.4 and 8.2.

### 2.3. Chemical Analysis

The concentrations of NH_4_^+^-N, NO_2_^−^-N, NO_3_^−^-N, MLSS, and MLVSS were measured according to standard methods (APHA, 1995). Total nitrogen (TN) was defined as the sum of NH_4_^+^-N, NO_2_^−^-N, and NO_3_^−^-N. Dissolved oxygen (DO) concentrations and pH values in the reactor were determined using a portable analyzer (HQ30d, Hach, USA) and a pH meter (PHS-10, Fangzhou, China). Nitrite accumulation ratio (NAR) and free ammonia (FA) were calculated as follows [30]:

(1) NAR%=NO2−-NEffNO2−-NEff+NO3−-NEff×100%

(2) FA  mgL=1714×NH4+-N+NH3-Nmg/L×10pHe6334/273+T+10pH.

### 2.4. Batch Tests

**Figure 1:**
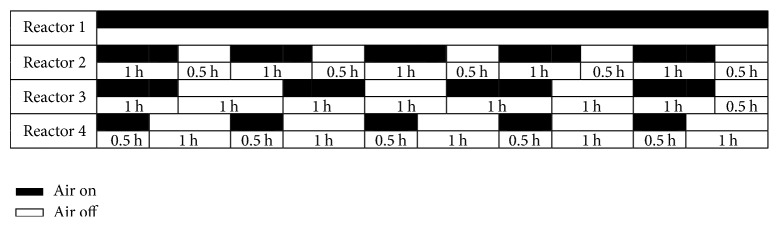
Aerated and nonaerated durations in batch reactors.

Tests to determine the effect of different aeration strategies (including continuous and intermittent aeration) on process efficiency were performed in batch reactors inoculated with the mixed liquor collected from the SBR prior to the end of the reaction period during the quasi-steady state. Tested sludge was washed to remove nitrogen compounds and was divided into four parts. Each part of the sludge was put into a 1.5 L batch reactor. Four batch reactors were supplied with synthetic wastewater. The initial NH_4_^+^-N concentration in four batch reactors was controlled at 280 mg N/L by adding equal amounts of ammonium bicarbonate. The first batch reactor received continuous aeration, and the remaining three all received intermittent aeration with different ratios of aerated to nonaerated duration: 0.5 h (aerated duration) to 1.5 h (nonaerated duration) for the second batch reactor, 1.0 h (aerated duration) to 1.0 h (nonaerated duration) for the third batch reactor, and 1.0 h (aerated duration) to 0.5 h (nonaerated duration) for the fourth batch reactor. The test duration was 450 min for each batch reactor. The aeration and nonaeration durations are depicted in Figure 1. pH was not controlled during the tests, and temperature was controlled at 30 ± 1°C.

### 2.5. DNA Analysis

A sludge sample was taken from the reactor on day 100 used for analysis of the microbial community. Direct DNA extraction was implemented using a fast DNA spin kit (SK8233) for soil (Sangon, Shanghai) according to the manufacturer's instructions. Partial 16S rRNA genes were amplified by PCR with a eubacteria primer set, F357 (5′-CCTACGGGAGGCAGCAG-3′) and R518 (5′-ATTACCGCGGCTGCTGG-3′). A GC clamp (CGCCCGCCGCGCCCCGCGCCCGGCCCGCCGCCCCCGCCCC) was added to 5′ of the forward primer to improve the detection of sequence variations in amplified DNA fragments by DGGE. The PCR reaction mixture was 50 mL and contained 5 mL of 10 × PCR buffer (Mg^2+^ plus), 1 mL of dNTP (10 mm L^−1^), 1 mL of each primer (10 mm L^−1^), 0.25 mL of Taq enzyme (5 U mL^−1^), and 0.5 mL of template DNA. The PCR reaction was carried out according to the following conditions: 4 min initial denaturation at 94°C, 30 cycles of 30 sec at 94°C, 60 sec at 56°C, 30 sec at 72°C, and 7 min final elongation at 72°C. The products were later resolved by denaturing gradient gel electrophoresis (DGGE) for 16 h at a constant voltage of 60 V at 60°C using the DCode system (Bio-Rad, Hercules, USA). An 8% polyacrylamide gel with a 30%–60% denaturing gradient was used to separate the PCR products (7 mol L^−1^ urea and 40% formamide comprised of 100% denaturant). Prominent DGGE bands were excited from the gel and dissolved in a 50 mL ITE buffer solution at 4°C, which was then reamplified as a template using the same methods previously described. The DNA fragments were later excited and reamplified using the primer sets F357 (without GC clamp) and R518, and the products were purified using the purification kit (Tiangen, China) and cloned using the pMD19-T plasmid vector system (TaKaRa, Japan). Sequencing was performed using an ABI 3730 DNA sequencer by a commercial service (Sangon, China). All sequences achieved were compared with similar sequences of the reference organisms using a BLAST search.

## 3. Results and Discussion

### 3.1. Performance of SBR

**Figure 2:**
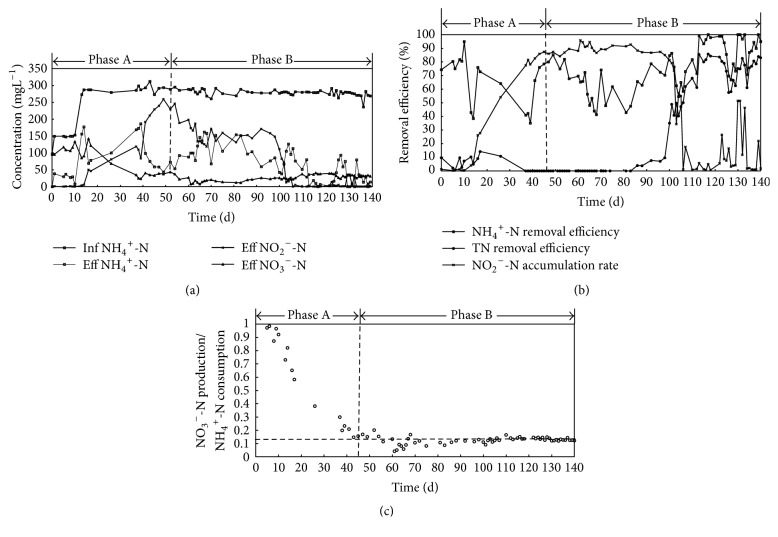
Performance of the CANON reactor: (a) influent and effluent concentrations of nitrogen species; (b) ammonium, TN removal efficiency, and nitrite accumulation rate (NAR); (c) ratio of nitrate production to ammonium consumption.

The reactor was operated in two phases over a time interval of 124 days. Each phase was characterized by the aeration mode and hydraulic retention time (HRT). Parameter settings of the different phases are summarized in Table 1. The concentrations of nitrogen compounds in the influent and effluent are depicted in Figure 2(a). The removal efficiency of ammonia nitrogen and total nitrogen (TN) and the nitrite accumulation ratio in effluent (i.e., the ratio of nitrite to the sum of nitrite nitrogen and nitrate nitrogen in effluent) are depicted in Figure 2(b). The ratios of nitrate production to ammonium consumption during start-up are shown in Figure 2(c). The solids retention time (SRT) was on average 120 days throughout the experiment. The biomass concentration was on average 2.14 g/L, and the ratio of VSS to TSS was 0.80 on average.

During phase A, three cycles were performed each day, and the continuous aeration mode was applied. In the first 10 days, the DO level in the bulk liquid was maintained between 1 and 2 mg/L, and the ammonia concentration in the influent was 150 mg/L. Figure 2(a) illustrates that the NH_4_^+^-N removal efficiency increased from 74.0% to 94.9% and that the nitrite concentration in the effluent was in the range of 0.24 to 1.16 mg/L. This finding indicates that ammonia oxidizers and nitrite oxidizers were cultivated in the SBR and converted the ammonia to nitrate via nitrite. To achieve partial nitrification in the system during days 11–50, DO concentrations were diminished, high free ammonia (FA) concentrations were maintained in the SBR, the mean DO concentration was fixed at 0.17 mg/L, and the mean FA concentration was increased to 7.36 mg/L. Under these conditions, nitrite concentration began to increase in the effluent due to DO limitation of the NOB and the inhibition of free ammonia, which when presented to NOB has characteristic lower oxygen affinities and higher sensitivities to free ammonia inhibition [31]. During this period, the removal efficiency of NH_4_^+^-N fluctuated in the range of 35.0% to 85.2%, while the nitrite accumulation ratio in effluent increased from 4.0% to 87.3%, as shown in Figure 2(b). This demonstrates that partial nitrification was achieved in the SBR at the end of phase A.

During phase B, two cycles were performed each day and intermittent aeration was applied from day 51 to day 123, after which three cycles were applied again to increase the nitrogen load of the SBR. The DO concentration in the aerated portion of the SBR cycle was maintained in the range of 0.04~0.15 mg/L. The removal of TN was minimal and was increased slowly between days 51 and 98. On the 99th day, 0.15 g of CANON biofilm biomass was taken into the SBR, after which the TN removal efficiency increased obviously, exceeding 80% on day 113 (14 days after CANON biofilm inoculation). The ratio of nitrate production to ammonium consumption was approximately 0.13, which one would expect for the CANON process [9] in the end of period B. This finding demonstrates the successful start-up of the CANON process in the SBR and indicates that the inoculation of AAOB biomass can significantly shorten the start-up time of the CANON process. Low DO concentrations and intermittent DO modes also played important roles in the realization of autotrophic nitrogen removal via nitrite.

### 3.2. Aeration Strategies

**Figure 3:**
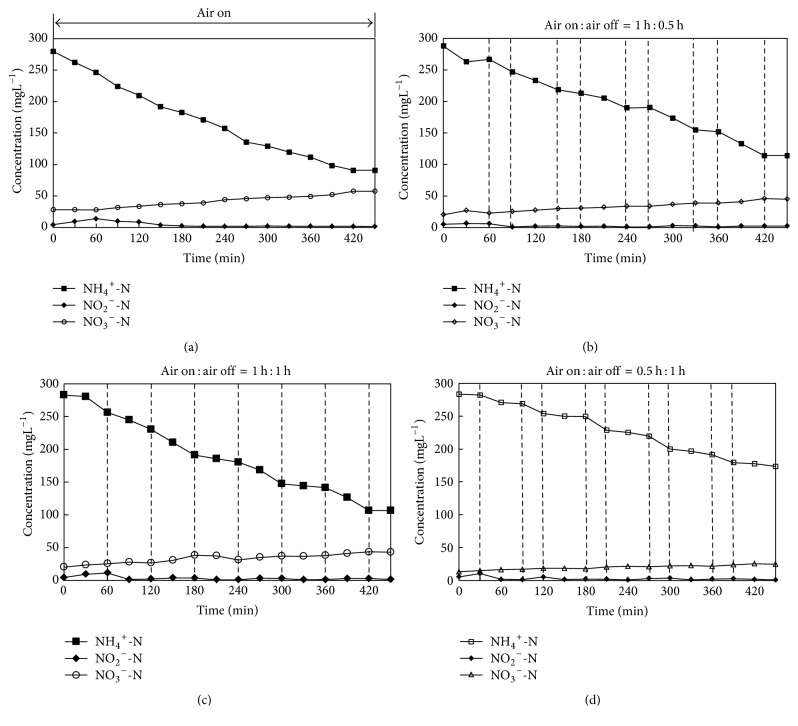
Profiles of nitrogen compounds in bulk solutions for a typical reaction time (450 min) of the SBR with four aeration strategies: (a) continuous aeration; (b) aeration time : nonaeration time = 1 h : 0.5 h; (c) aeration time : nonaeration time = 1 h : 1 h; (d) aeration time : nonaeration time = 0.5 h : 1 h.

Changes of nitrogen compounds under different aeration strategies are depicted in Figures 3(a), 3(b), 3(c), and 3(d). It is demonstrated in Figure 3(a) that ammonia concentrations decreased almost linearly under continuous aeration. Nitrite concentration increased in the first 60 min, later decreased, and subsequently remained steady between 1.5 and 2.0 mg/L. Nitrate concentration increased from 28.33 mg/L to 57.48 mg/L. The removal efficiency of ammonia was 73.72%, the total nitrogen removal efficiency was 65.56%, and the ratio of nitrate production to ammonium consumption was 0.154, which was much higher than what one would expect for the CANON system [9]. The second aeration strategy included 5 aeration times and 5 nonaeration times (Figure 1), where each aeration time lasted 60 min and each nonaeration time lasted 30 min. The nitrogen compounds of the second aeration strategy are shown in Figure 3(b). It can be seen that ammonia decreased in each aeration time, while the variation of ammonia in each nonaeration time differed. Ammonia concentration decreased in the first two nonaeration times but remained unchanged in the following three nonaeration times. Additionally, it was observed that nitrite concentrations were very low in the subsequent three nonaeration times, which limited the activity of AAOB and resulted in unchanged ammonia concentrations. The total nitrogen removal efficiency of the second aeration strategy was 67.02%, and the ratio of nitrate production to ammonia consumption was 0.137, which was still higher than what one would expect for the CANON system. There were 4 aeration times and 4 nonaeration times in the third aeration strategy. All aeration times were 1 h, and nonaeration times were 1 h, 1 h, 1 h, and 0.5 h. Variation of nitrogen compounds used in the third aeration strategy was similar to those used in the second aeration strategy. The ammonia and total nitrogen removal efficiencies of the third aeration strategy were 62.93% and 69.21%, respectively. The ratio of nitrate production to ammonium consumption was 0.128, which was closest to what one would expect for the CANON system in the four batch tests. The fourth aeration strategy also included 5 aeration times and 5 nonaeration times, and the ratio of aeration time to nonaeration time was 0.5 h : 1 h. The ammonia and total nitrogen removal efficiencies of the fourth strategy were only 49.83% and 54.22%, respectively, due to the aeration times being too short to convert enough ammonia to nitrite. The activity of AAOB was also limited by the lack of nitrite.

The total aeration times in the four aeration strategies were 450 min (continuous aeration), 300 min (aeration time : nonaeration time = 1 h : 0.5 h), 240 min (aeration time : nonaeration time = 1 h : 1 h), and 150 min (aeration time : nonaeration time = 0.5 h : 1 h). The total nitrogen removal efficiencies of the first, second, third, and fourth strategies were 65.56%, 62.93%, 65.33%, and 54.22%, respectively. The ratios of nitrate production to ammonia consumption for the first, second, third, and fourth strategies were 0.154, 0.142, 0.128, and 0.103, respectively. This finding suggests that the ammonia conversion rate and the nitrate production rate decreased with decreasing aeration times, which were also related to the activities of AOB, NOB, and AAOB. The CANON process relied on the incorporation of AOB and AAOB, while NOB was not expected in the reactor. The ratio of nitrate production to ammonia consumption was 0.13 when the NOB had washed out completely [9]. A ratio higher than 0.13 indicated that NOB was not washed out completely from the SBR. The activity of AAOB was inhibited by oxygen when oxygen penetrated into the inner of floc in the aeration duration, while the nonaeration duration can not only inhibit the activity of NOB but also help the AAOB to restore its activity, which was inhibited by oxygen in the aeration duration. The strategy of 0.5 h aeration and 1 h nonaeration is not recommended because the short aeration time limited the production of nitrite, resulting in ammonia and total nitrogen removal efficiencies being very low. The very low ratio of nitrate production to ammonia consumption further verified that the short aeration time limited the conversion of ammonia. The strategy of 1 h aeration and 1 h nonaeration was the optimal one, which resulted in similar total nitrogen removal and continuous aeration efficiency and shortened the duration of aeration. The ratio of nitrate production to ammonia consumption under this strategy was also close to the one expected for the CANON system [9].

### 3.3. Biomass Morphology

**Figure 4:**
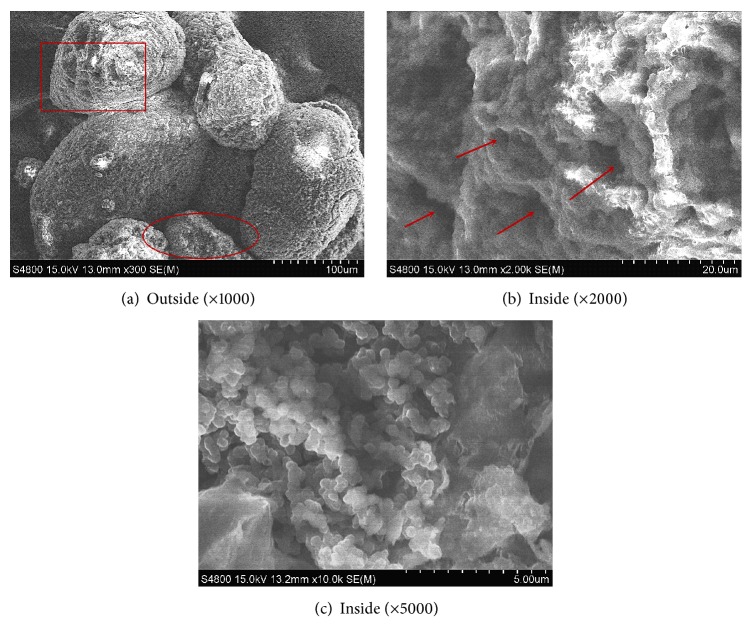
Scanning electron microscopy (SEM) images of granules.

At the start of this experiment, 1 liter of activated sludge from a municipal WWTP was taken into the SBR, and a small CANON biofilm was inoculated in the SBR after the partial nitrification was set up. Over time, the granular sludge started to grow in the SBR. At the end of the experiment, the sludge in the SBR was a mixture of floc and granules, with the granules occupying 31% (w/w) and the flocs occupying 69% (w/w). Three scanning electron microscopy (SEM) images of the granules are shown in Figure 4: (a) the outside of a granule, (b) one slice of a granule (a rectangle), and (c) another slice of a granule (an ellipse). Arrows in Figure 4(b) indicated holes inside the granule. Granules had distinct boundary and had spherical or ellipsoidal appearance, while flocs were more loose and amorphous. The average diameter of the granules was 900 *μ*m, while the size of floc was smaller than 300 *μ*m.

### 3.4. DNA Analysis

PCR-DGGE was used to examine the microbial community in the SBR, and parts of the results are shown in Table 2. Results show that six groups had high identity to* Candidatus Brocadia* belonging to the order Planctomycetales.* Candidatus Brocadia* was identified as the dominant species of AnAOB in the CANON process. This finding indicates that only one genus of anammox bacteria is dominant under the applied growth conditions [32]. Three groups of AerAOB belonging to *β*-proteobacteria were found in this reactor, all of which were closely related to* Nitrosomonas*, which have been found as dominant populations in other CANON systems [33, 34]. NOB belonging to* Nitrospira* was also present in the reactor, which further supports the results of batch tests. The NOB was present in floc when the DO was sufficient, producing nitrate and resulting in unstable performance of the CANON system.

## 4. Conclusions

Inoculating AAOB biomass in a partial nitrification system can start the CANON process rapidly, resulting in total nitrogen removal efficiencies of up to 80% after inoculation. The presence of complete granular sludge was not necessary. The mixture of granular sludge and floc present in the SBR and the NOB present in the floc and sludge influenced the total nitrogen removal efficiency. Granules had distinct boundary and were spherical or ellipsoidal, while floc was more loose and amorphous. The average diameter of the granules was 900 *μ*m, while the size of floc was smaller than 300 *μ*m. The intermittent aeration strategy was important not only for ammonia and total nitrogen efficiencies but also for the inhibition of NOB and the protection of AAOB from oxygen. The optimal ratio of aeration time to nonaeration time for the intermittent aeration strategy was 1 h : 1 h.

## Figures and Tables

**Table 1:** Operating parameters of each phase in SBR.

Parameters	Phase A	Phase B
Time range (d)	0~50	51~140

Hydraulic retention time (h)	16	24 (51~123 days), 16 (124~140 days)

Aeration mode	Continuous	Intermittent (2 h aeration : 2 h no aeration : 2 h aeration : 1.5 h no aeration)

DO concentration during aeration phase	1~2 mg/L (1~11 days), 0.16~0.18 mg/L (12~51 days)	0.04~0.15 mg/L (aeration time), 0 mg/L (no aeration time)

Intermittent aeration (R)	—	1/1 (except the last nonaeration duration)

**Table 2:** Phylogenetic sequence similarities to the closest relative from DGGE bands.

Band	Taxon	Identity	Accession	Phylum (classifier)
4	*Candidatus Brocadia *sp. RAS-Ina-1 16S rRNA gene	97%	HM769652	Planctomycetia
5	*Candidatus Brocadia *sp. S08-01 16S rRNA gene	99%	JN205347	Planctomycetia
7	*Candidatus Brocadia fulgida* isolate R1 16S rRNA gene	97%	JQ864321	Planctomycetia
15	*Candidatus Brocadia *sp.ODS-1 16S rRNA gene	98%	HM769653	Planctomycetia
19	Uncultured *Candidatus Brocadia *sp.16S rRNA gene	98%	KF606756	Planctomycetia
23	*Candidatus Brocadia caroliniensis *16S rRNA gene	98%	KF810110	Planctomycetia
1	Uncultured *Nitrosomonas *sp. gene for 16S rRNA gene	100%	AB500059	*β*-proteobacteria
26	*Nitrosomonas *sp. HP8 partial 16S rRNA gene	99%	HF678378	*β*-proteobacteria
28	Uncultured *Nitrosomonas *sp.DGGE 16SRNA gene	100%	KF452288	*β*-proteobacteria
2	*Nitrospira*	98%	KF472236	*β*-proteobacteria

## References

[1] Lackner S., Gilbert E. M., Vlaeminck S. E., Joss A., Horn H., van Loosdrecht M. C. M. (2014). Full-scale partial nitritation/anammox experiences—an application survey. *Water Research*.

[2] Vázquez-Padín J., Mosquera-Corral A., Campos J. L., Méndez R., Revsbech N. P. (2010). Microbial community distribution and activity dynamics of granular biomass in a CANON reactor. *Water Research*.

[3] Mulder A., van de Graaf A. A., Robertson L. A., Kuenen J. G. (1995). Anaerobic ammonium oxidation discovered in a denitrifying fluidized bed reactor. *FEMS Microbiology Ecology*.

[4] Strous M., Heijnen J. J., Kuenen J. G., Jetten M. S. M. (1998). The sequencing batch reactor as a powerful tool for the study of slowly growing anaerobic ammonium-oxidizing microorganisms. *Applied Microbiology and Biotechnology*.

[5] Güven D., Van De Pas-Schoonen K., Schmid M. C. (2004). Implementation of the anammox process for improved nitrogen removal. *Journal of Environmental Science and Health, Part A: Toxic/Hazardous Substances and Environmental Engineering*.

[6] Chamchoi N., Nitisoravut S. (2007). Anammox enrichment from different conventional sludges. *Chemosphere*.

[7] Molinuevo B., García M. C., Karakashev D., Angelidaki I. (2009). Anammox for ammonia removal from pig manure effluents: Effect of organic matter content on process performance. *Bioresource Technology*.

[8] Valverde-Pérez B., Mauricio-Iglesias M., Sin G. (2016). Systematic design of an optimal control system for the SHARON-Anammox process. *Journal of Process Control*.

[9] Third K. A., Sliekers A. O., Kuenen J. G., Jetten M. S. M. (2001). The CANON system (completely autotrophic nitrogen-removal over nitrite) under ammonium limitation: interaction and competition between three groups of bacteria. *Systematic and Applied Microbiology*.

[10] Sliekers A. O., Third K. A., Abma W., Kuenen J. G., Jetten M. S. M. (2003). CANON and Anammox in a gas-lift reactor. *FEMS Microbiology Letters*.

[11] Windey K., De Bo I., Verstraete W. (2005). Oxygen-limited autotrophic nitrification-denitrification (OLAND) in a rotating biological contactor treating high-salinity wastewater. *Water Research*.

[12] Hao X., Heijnen J. J., Van Loosdrecht M. C. M. (2002). Model-based evaluation of temperature and inflow variations on a partial nitrification-ANAMMOX biofilm process. *Water Research*.

[13] Sliekers A. O., Derwort N., Campos Gomez J. L., Strous M., Kuenen J. G., Jetten M. S. M. (2002). Completely autotrophic nitrogen removal over nitrite in one single reactor. *Water Research*.

[14] Schmid M., Walsh K., Webb R. (2003). Candidatus "Scalindua brodae", sp. nov., Candidatus "Scalindua wagneri", sp. nov., Two New Species of Anaerobic Ammonium Oxidizing Bacteria. *Systematic and Applied Microbiology*.

[15] Trigo C., Campos J. L., Garrido J. M., Méndez R. (2006). Start-up of the Anammox process in a membrane bioreactor. *Journal of Biotechnology*.

[16] Cho S., Fujii N., Lee T., Okabe S. (2011). Development of a simultaneous partial nitrification and anaerobic ammonia oxidation process in a single reactor. *Bioresource Technology*.

[17] Liu S., Yang F., Gong Z., Su Z. (2008). Assessment of the positive effect of salinity on the nitrogen removal performance and microbial composition during the start-up of CANON process. *Applied Microbiology and Biotechnology*.

[18] Pynaert K., Smets B. F., Beheydt D., Verstraete W. (2004). Start-up of autotrophic nitrogen removal reactors via sequential biocatalyst addition. *Environmental Science & Technology*.

[19] Gong Z., Yang F., Liu S., Bao H., Hu S., Furukawa K. (2007). Feasibility of a membrane-aerated biofilm reactor to achieve single-stage autotrophic nitrogen removal based on Anammox. *Chemosphere*.

[20] Lv Y., Wang L., Sun T., Wang X., Yang Y., Wang Z. (2010). Autotrophic nitrogen removal discovered in suspended nitritation system. *Chemosphere*.

[21] Vlaeminck S. E., Terada A., Smets B. F. (2010). Aggregate size and architecture determine microbial activity balance for one-stage partial nitritation and anammox. *Applied and Environmental Microbiology*.

[22] Nielsen M., Bollmann A., Sliekers O. (2005). Kinetics, diffusional limitation and microscale distribution of chemistry and organisms in a CANON reactor. *FEMS Microbiology Ecology*.

[23] Arrojo B., Mosquera-Corral A., Campos J. L., Méndez R. (2006). Effects of mechanical stress on Anammox granules in a sequencing batch reactor (SBR). *Journal of Biotechnology*.

[24] Schaubroeck T., Bagchi S., De Clippeleir H., Carballa M., Verstraete W., Vlaeminck S. E. (2012). Successful hydraulic strategies to start up OLAND sequencing batch reactors at lab scale. *Microbial Biotechnology*.

[25] Vangsgaard A. K., Mutlu A. G., Gernaey K. V., Smets B. F., Sin G. (2013). Calibration and validation of a model describing complete autotrophic nitrogen removal in a granular SBR system. *Journal of Chemical Technology and Biotechnology*.

[26] Vlaeminck S. E., Cloetens L. F. F., De Clippeleir H., Carballa M., Verstraete W. (2009). Granular biomass capable of partial nitritation and anammox (Water Science and Technology 58(5) 1113-1120). *Water Science and Technology*.

[27] Lemaire R., Webb R. I., Yuan Z. (2008). Micro-scale observations of the structure of aerobic microbial granules used for the treatment of nutrient-rich industrial wastewater. *The ISME Journal*.

[28] Scuras S., Daigger G. T., Grady C. P. L. (1998). Modeling the activated sludge floc microenvironment. *Water Science and Technology*.

[29] Yang J., Trela J., Zubrowska-Sudol M., Plaza E. (2015). Intermittent aeration in one-stage partial nitritation/anammox process. *Ecological Engineering*.

[30] Anthonisen A. C., Loehr R. C., Prakasam T. B. S., Srinath E. G. (1976). Inhibition of nitrification by ammonia and nitrous acid. *Journal of the Water Pollution Control Federation*.

[31] van der Star W. R. L., Miclea A. I., van Dongen U. G. J. M., Muyzer G., Picioreanu C., van Loosdrecht M. C. M. (2008). The membrane bioreactor: a novel tool to grow anammox bacteria as free cells. *Biotechnology and Bioengineering*.

[32] Hu B.-L., Zheng P., Tang C.-J. (2010). Identification and quantification of anammox bacteria in eight nitrogen removal reactors. *Water Research*.

[33] Liu S., Yang F., Xue Y. (2008). Evaluation of oxygen adaptation and identification of functional bacteria composition for anammox consortium in non-woven biological rotating contactor. *Bioresource Technology*.

[34] Liu T., Li D., Zeng H. (2012). Biodiversity and quantification of functional bacteria in completely autotrophic nitrogen-removal over nitrite (CANON) process. *Bioresource Technology*.

